# Lens Coloboma: A Rare Association of Congenital Rubella Syndrome

**DOI:** 10.7759/cureus.39355

**Published:** 2023-05-22

**Authors:** Vinita Gupta, Aarshi Naharwal, Pallavi Sharma, Saurabh Luthra

**Affiliations:** 1 Ophthalmology, All India Institute of Medical Sciences, Rishikesh, Rishikesh, IND; 2 Ophthalmology, Drishti Eye Institute, Dehradun, IND

**Keywords:** retinal detachment, bilateral cataracts, high myopia, lens coloboma, crs, congenital rubella syndrome

## Abstract

Congenital rubella syndrome (CRS) may affect all ocular structures in general, either in isolation or in combination. Typical ocular complications in CRS include cataracts, microcornea, microphthalmia, glaucoma, nystagmus, and retinopathy. We report a case of a four-year-old girl who presented with bilateral total cataracts with sensory nystagmus, a poorly dilating pupil, iris hypoplasia, high axial myopia in the right eye (RE), and a retinal detachment in the left eye (LE). The systemic evaluation revealed microcephaly with an associated patent ductus arteriosus (PDA) and mild pulmonary arterial hypertension (PAH). Based on these findings, the child was diagnosed with clinically confirmed CRS. The child was taken up for right-eye cataract surgery. Intra-operatively, a lens coloboma involving the temporal equator of the lens along with a highly tessellated fundus was noted. Two weeks post-cataract surgery, the child had a best corrected visual acuity (BCVA) of 6/24. However, four weeks after the surgery, the child developed total rhegmatogenous retinal detachment in the right eye, for which pars plana vitrectomy with endolaser and silicon oil tamponade was done. Four weeks later, the child’s BCVA in the right eye was 6/36, which was maintained until the last follow-up of four months. Lens coloboma may be isolated or may occur in association with chorioretinal coloboma. Ours is the first case of unilateral atypical lens coloboma associated with high myopia and bilateral cataracts in a patient with congenital rubella syndrome. Lens colobomas with high myopia have not been reported previously as ocular associations of CRS. Our case highlights that in children with CRS presenting with unilateral or bilateral congenital cataracts, the possibility of lenticular coloboma as a coexistent association should be kept in mind while taking these cases for cataract surgery.

## Introduction

Women infected with the rubella virus have a very high chance of passing the virus to their foetus during the early stages of pregnancy, which may result in the death of the foetus or the development of congenital rubella syndrome (CRS). CRS presents with a wide spectrum of ocular and systemic abnormalities, including sensorineural hearing loss, cardiovascular defects, neurological anomalies, and hepato-splenomegaly [1]. Ocular manifestations of CRS include cataracts (93.1%), microphthalmos (85.1%), iris abnormalities (58.6%), pigmentary retinopathy (37.9%), strabismus (26%), nystagmus (50%), glaucoma (6%), optic atrophy (4.6%), and congenital dacryocystitis (2.3%). Among these, a combination of cataracts, microphthalmos, and iris hypoplasia is the most common presentation in about 56% of cases [2]. Lens coloboma, characterized by a straight edge or notch at the lens equator, occurs because of localized zonular absence. The shape and size of coloboma vary. Lens coloboma may occur anywhere along the circumference of the lens but typically occurs in the downward position. It is usually unilateral and single but may be bilateral and double. It may be seen as an isolated anomaly in one eye or may occur bilaterally in association with colobomas of the uveal system [3]. Association with other ocular abnormalities such as retinal detachment, optic disc coloboma, or hypoplasia has also been observed [4-5]. While lens coloboma may not be accompanied by systemic findings, isolated lens coloboma has been seen in some systemic disorders such as Marfan’s syndrome, Marshall’s syndrome, and Alport-like glomerulonephritis [6-9]. Herein, we report a case of unilateral atypical lens coloboma associated with high axial myopia and bilateral cataracts in a patient with congenital rubella syndrome.

## Case presentation

**Figure 1:**
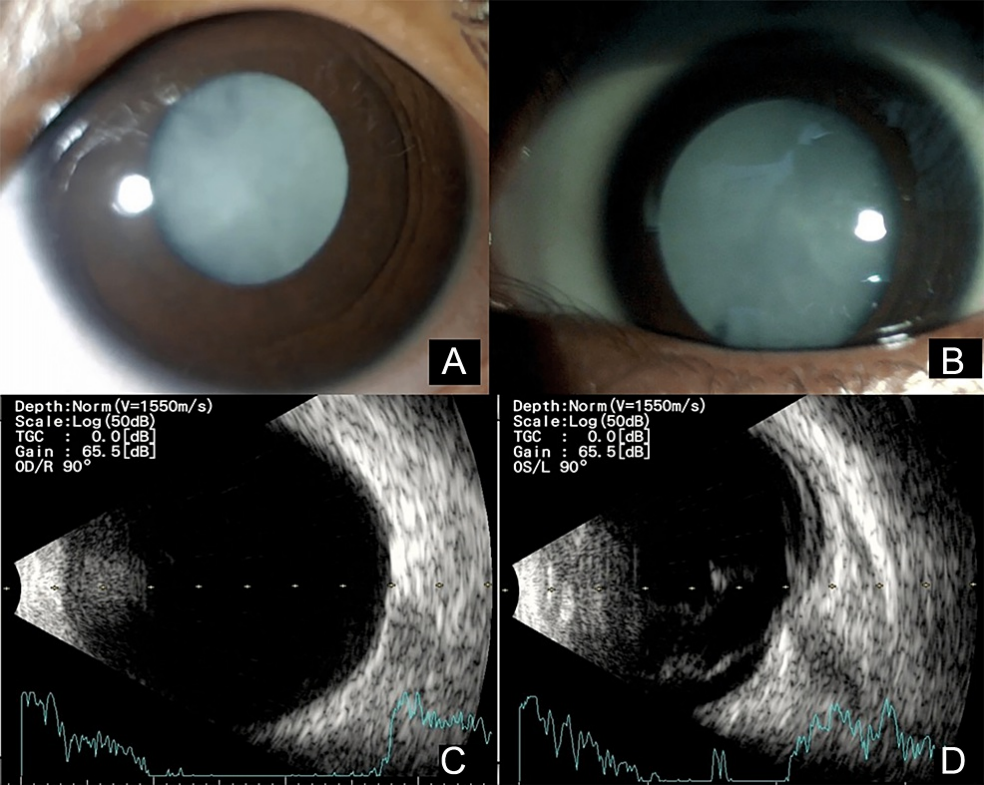
Pre-operative photographs of both eyes. (A) Anterior segment photograph of right eye with poorly dilating pupil and total cataract; (B) anterior segment photograph of left eye with well dilated pupil and total cataract; (C) ultrasound-B scan of right eye showing echo free vitreous with attached retina; (D) ultrasound-B scan of left eye showing moderate to high reflective membranous vitreous echoes with restricted after movements suggestive of retinal detachment.

A four-year-old girl presented to us with a white reflex in both eyes (BE), noticed by her parents when she was two months old. Birth history was uneventful, and all developmental milestones were normal for age. The child had a vision of light perception in the right eye (RE) and no perception of light in the left eye (LE). Ocular examination revealed bilateral total cataracts with sensory nystagmus, a poorly dilating pupil, and iris hypoplasia in the RE (Figure 1A-1B), with a relative afferent pupillary defect in the LE. B-scan ultrasonography showed an echo-free vitreous cavity with an attached retina in the RE (Figure 1C), while the LE had moderate to high reflective membranous echoes in the vitreous with restricted after movements suggestive of retinal detachment (Figure 1D). The axial length of RE was 28.85 mm and that of LE was 22.77 mm. A systemic evaluation revealed microcephaly without any developmental delays or neurological deficits. There was an association between patent ductus arteriosus (PDA) and mild pulmonary arterial hypertension (PAH). Pure-tone audiometry was bilaterally normal. The patient was diagnosed with clinically confirmed congenital rubella syndrome with microcephaly, PDA, PAH, and both eyes total cataracts with sensory nystagmus, RE iris hypoplasia, and LE retinal detachment. Laboratory investigations revealed high rubella IgG avidity, suggesting a remote rubella infection.

**Figure 2:**
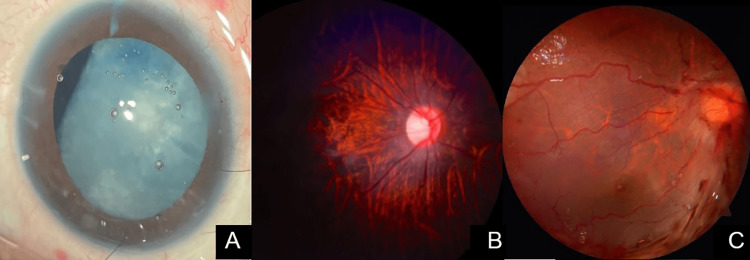
(A) Intra-operative photograph of right eye showing absent zonules in the temporal quadrant extending from 11 o' clock to 5 o' clock with specks of calcifications on anterior lens capsule; (B) post-operative fundus photograph of right eye after cataract surgery showing highly tessellated fundus and chorioretinal atrophic patches; (C) post-operative fundus photograph of right eye after retinal detachment surgery showing silicon oil filled eye with attached retina.

The patient was planned for RE cataract surgery with a poor prognostication for LE surgical intervention. The intraocular pressure recorded with Perkin’s tonometer under anaesthesia was 14 mm Hg in the RE and 8 mm Hg in the LE. Intra-operatively, after intra-ocular instillation of adrenaline and viscoelastic RE, the pupil dilated well, and a lens coloboma involving the temporal quadrant extending from 7 to 11 o'clock with a calcified anterior lens capsule was noted in the RE (Figure 2A). An anterior continuous curvilinear capsulorrhexis was initiated; however, it could not be completed due to poor zonular support and a calcified anterior capsule. Hence, lensectomy-anterior vitrectomy was done with a vitrectomy cutter without intra-ocular lens (IOL) implantation. Retinal examination at the end of surgery revealed a tesselated fundus with chorioretinal atrophic patches and no treatable lesion in the peripheral retina of the RE (Figure 2B). In the immediate post-operative period, RE had the best corrected visual acuity (BCVA) of 6/24 (+5.00 DS), which was maintained for two weeks. She was planning for secondary IOL implantation. However, the child presented again two weeks later with a sudden diminution of vision in the operated eye. This diminution of vision was due to total rhegmatogenous retinal detachment with a rhegma at 9 o'clock in the temporal mid periphery, for which pars plana vitrectomy with endolaser with silicon oil tamponade was done (Figure 2C). Post-operative BCVA in the RE four weeks later was 6/36 (+3.00 DS/−4.00 DC X 450), which was maintained till the last follow-up of four months.

## Discussion

Rubella virus infection, in most cases, is a mild disease but can cause severe defects in the developing foetus and newborn from maternal infection during pregnancy (CRS). During the first two months of gestation, the foetus has a 65% to 85% chance of being affected, with an outcome of multiple congenital defects or spontaneous abortion [10]. The classic triad of clinical manifestations associated with CRS among surviving neonates is hearing impairment, congenital heart defects, in particular branch pulmonary artery stenosis and patent ductus arteriosus, and eye anomalies such as cataract, salt and pepper type pigmentary retinopathy, chorioretinitis, or congenital glaucoma. Postnatal diagnosis of congenital rubella infection is done by detecting rubella virus-IgG antibodies in neonatal serum using an enzyme-linked immunosorbent assay. This approach has a sensitivity and specificity of nearly 100% in infants less than three months of age. Rubella reverse transcription polymerase chain reaction (RT-PCR) assays on throat swabs, nasopharyngeal swabs, and urine specimens from a neonate can be used for confirmation of suspected CRS cases [11-12]. Nonetheless, its utility is limited because of the narrow window when the virus can be detected in clinical samples. Congenital infection can also be confirmed by stable or increasing serum concentrations of rubella-specific IgG over the first year of life. However, it is difficult to diagnose congenital rubella in children older than one year of age [13].

Measurement of rubella IgG antibody avidity can be used to distinguish between recent exposure to rubella and more distant rubella exposure. Low-avidity rubella IgG suggests a recent infection and can be detected for up to four months after infection. High avidity rubella IgG suggests a more distant rubella exposure, which may be from either infection or vaccination [11]. Our child had PDA, PAH with microcephaly, and both eyes had total cataracts. He was diagnosed with clinically confirmed CRS as per the CRS case definition by the Center for Disease Control and Prevention [14]. Our case had clinical features suggestive of CRS and high rubella IgG avidity.

Ocular coloboma is a well-known entity with an estimated prevalence of 0.7 in 10,000 in the world population [15]. It develops because of the failure of the embryonic fissure to close at either end, which results in a spectrum of corneal, iris, ciliary body, choroidal, retinal, and optic nerve defects depending on the end where the embryonic fissure fails to close. Lens coloboma occurs at about the fourth month of development and is divided into typical (those that occur at the site of the embryonic fissure) and atypical (those that do not occur at the site of the embryonic fissure) [16]. In the region of coloboma, the zonules are either absent or maldeveloped. Toxic, inflammatory, and genetic factors have been implicated as factors responsible for the maldevelopment/absence of zonules in the equatorial area of the lens [17]. In cases of typical colobomas, maldevelopment may be due to incomplete closure and development of the optic vesicles, and typical colobomas are hence associated with other colobomas in the eye [3].

Lens coloboma may cause a diminution of vision either due to lenticular astigmatism or associated ocular abnormalities such as cataracts, high myopia, or fundal coloboma [17]. It may also cause amblyopia if it is unilateral [18]. Our child had an axial length as measured by ultrasonic biometry of 28.85 mm in RE and 22.77 mm in LE, indicative of high axial myopia in RE. She was likely to be high myopic in LE as well; the shorter AL of 22.77 mm could have been fallaciously recorded because of low intra-ocular pressure secondary to retinal detachment. The association of retinal detachment with lens coloboma has been described earlier. Hovland et al. have reported bilateral retinal detachment in eight cases of nasal coloboma of the lens [4]. The retinal detachments in their cases were secondary to giant retinal breaks and appeared to be related to abnormal differentiation of the tertiary vitreous in the third to fourth month of gestation. Except for one case that had aminoaciduria (the aetiology of which was not described), no other case had any systemic association. Bavbek et al. have also reported two cases of unilateral lens coloboma with a retinal detachment that were associated with retino-choroidal coloboma in one and retinoschisis in the other without any systemic association [17]. Our case had a unilateral temporal lens coloboma associated with high axial myopia, bilateral cataracts, and retinal detachment. Lens coloboma in our child could be attributed to the toxic effect of the rubella virus on the development of zonules, while retinal detachment could have been associated with coloboma or high myopia. Microphthalmos has been described as having an ocular association with CRS [2]. Microphthalmic eyes are usually hypermetropic but can sometimes be highly myopic if there is a staphyloma [19]. Our child had high axial myopia in the right eye without any retinochoroidal colobomas or posterior staphylomas.

To the best of our knowledge, ours is the first case of unilateral atypical lens coloboma with high myopia associated with bilateral cataracts in a patient with congenital Rubella syndrome.

## Conclusions

Patients with congenital rubella syndrome have cataracts, microphthalmos, and iris hypoplasia as the most common ocular manifestations. Isolated lens colobomas without other retinochoroidal colobomas have been reported in systemic disorders like Marfan's syndrome, Marshall’s syndrome, and Alport-like glomerulonephritis. However, they may also occur in cases of congenital rubella syndrome, as seen in our case.
